# Retraction Notice

**DOI:** 10.2144/fsoa-2020-0006r1

**Published:** 2020-06-25

The following article has been retracted from *Future Science OA* due to substantial overlap with a previously published article:

Retracted article: Beri K. Exploring the microbiome and mindfulness connection. *Future Sci. OA* doi: 10.2144/fsoa-2020-0006 (2020).

Previous article: Schnorr SL, Bachner HA. Integrative therapies in anxiety treatment with special emphasis on the gut microbiome. *Yale J. Biol. Med.* 89(3), 397–422 (2016).

The author and editors of *Future Science OA* regret any negative consequences this publication might have caused in the scientific and medical communities.

